# Overweight and Obesity and Their Association with Neurodevelopmental Delay Among Preschoolers

**DOI:** 10.3390/epidemiologia7050128

**Published:** 2026-09-16

**Authors:** Ana Cristina Castañeda-Márquez, Yolanda Martínez-López, Jaime Salvador-Moysén, Edgar Felipe Lares-Bayona, José Ángel Hernández-Mariano

**Affiliations:** 1 Scientific Research Institute, Juarez University of the State of Durango, Durango 34000, Mexico; 2 Department of Research, Hospital Juarez of Mexico, Mexico City 07760, Mexico

**Keywords:** obesity, overweight, children, neurodevelopmental delay

## Abstract

**Background/Objectives**: Neurodevelopmental delays can have significant long-term consequences. Given that overweight and obesity have been associated with alterations in several biological processes related to child development, we aimed to evaluate their association with neurodevelopmental delays in children. **Methods**: We conducted an analytical cross-sectional study among 280 children aged 2–5 years. Child neurodevelopmental information was obtained from the Battelle Developmental Inventory Screening Test, which includes 96 items and measures fundamental developmental skills in five domains: Personal/Social; Adaptive; Motor; Communication; and Cognitive. Nutritional status was obtained from weight and height measurements. Anthropometric indicators, including BMI and height-for-age, were calculated. World Health Organization (WHO) cutoff points were used to classify children as overweight or obese. Associations were assessed using logistic regression models adjusted for confounders and evaluating interaction terms. **Results**: A total of 19.8% were overweight or obese. More than 20% of the children showed delays in at least one developmental domain. Participants who were overweight or obese were more likely to have a delay in the Personal/Social domain (Odds Ratio (OR) = 2.36, *p* = 0.03). Children who were overweight or obese and whose mothers were ≤30 years old were more likely to have a delay in the Adaptive domain (OR = 4.8, *p* = 0.02); on the other hand, being male and being overweight or obese increased the likelihood of having a delay in the Personal/Social domain (OR = 11.1, *p* = 0.006). Similar results were observed in the communication domain (OR = 5.88, *p* = 0.007). **Conclusions**: The results suggest that nutritional status is associated with delays in Personal/Social, Adaptative and Communication domains; the child’s sex and the mother’s age may modify this association.

## 1. Introduction

Neurodevelopment is an organized and coordinated process in which the brain acquires an increasingly complex structure, reflected in new functional abilities, improved adaptive performance, and, finally, favorable human development [1]. Child development is recognized as a non-linear process, marked by fluctuating progress, with advances and setbacks, because the child’s brain at this stage is constantly developing and is influenced by biological, psychological, and social factors [2].

Developmental milestones enable us to objectively assess whether children are acquiring the skills expected for their age, facilitating ongoing monitoring of their development [3]; however, in some cases, children fail to reach these milestones, resulting in developmental delays [4]. In this regard, according to data from the World Health Organization (WHO), approximately 10% of each country’s population may have a neurodevelopmental disorder [5], and according to estimates made in 2016, approximately 52.9 million children worldwide had some type of developmental delay, being more frequent in developing countries [4]. In Latin America, estimates by some authors indicate that between 10 and 20% of the children studied presented neurodevelopmental delays [6,7], while in Mexico, estimates exceed 20% [8,9].

The neurodevelopmental process depends mainly on the genetic and environmental factors to which the child is exposed. In this context, adequate nutritional status is essential for this process to develop correctly, as deficiencies or excesses can cause various functional difficulties that negatively affect neurodevelopment [10].

Excess body weight may influence neurodevelopment through multiple biological and biomechanical pathways. From a motor perspective, increased body weight alters the center of gravity. It places greater mechanical stress on the musculoskeletal system, impairing posture, balance, and the execution of coordinated movements. At the same time, fatigue and reduced physical activity may further limit the sensorimotor experiences necessary for motor development [11,12]. At the cognitive level, obesity has been associated with chronic low-grade inflammation and increased adipokine production, which may affect brain regions involved in executive functions, learning, memory, and self-regulation. In addition, obesity-related sleep disturbances, such as obstructive sleep apnea, may further compromise neurodevelopment through intermittent hypoxia and impaired sleep quality [13,14]. Finally, excess weight may also influence social and emotional development by limiting participation in play and peer interaction, increasing the risk of stigma and social exclusion, particularly in obesogenic environments characterized by limited access to healthy foods and opportunities for physical activity [15].

In this regard, some authors have suggested an association between poor nutritional status and neurodevelopmental delays, particularly in the social, motor, and language areas [16,17]. On the other hand, similar associations have been reported with excessive nutritional status, that is, overweight or obesity [18,19,20]; nevertheless, this association has been little studied. In Mexico, childhood overweight and obesity represent a serious public health problem, which could have significant repercussions on neurodevelopment and, consequently, on the development of human potential. Because of this, it is essential to generate scientific evidence on the role that overweight or obesity plays in neurodevelopmental disorders. Therefore, we aimed to evaluate the association between overweight or obesity and neurodevelopmental delays among Mexican children.

## 2. Materials and Methods

### 2.1. Study Design and Setting

We conducted an analytical cross-sectional study involving 280 Mexican children aged 2 to 5 years. Participants were recruited from a public early childhood education and preschool center in Durango, Mexico. This public institution serves approximately 350 children daily from diverse socioeconomic backgrounds, offering both daycare and formal preschool education. Children attend the center primarily for early education, socialization, and preschool education while their parents are at work. The study included children who regularly attended the institution and showed no physical or neurological disabilities at the time of the assessment. Participants were selected using convenience sampling. The study population was recruited and assessed from October 2023 to June 2024.

The sample size calculation was performed using the formula for odds ratios (OR), aiming for an OR of at least 2.0, considering a prevalence of neurodevelopmental delays of 32.1% [9] and a statistical power of 80%, resulting in a minimum number of participants of 276 children.

### 2.2. Data Collection Tool and Technique

Data were collected via a structured survey comprising two sections. The first section was administered to the child’s parents or legal guardians and gathered sociodemographic information, including the child’s age, sex, type of delivery, birth weight, and gestational age at birth, as well as maternal variables such as age, educational level, marital status, employment status, and number of children. The second section assessed the child’s neurodevelopment.

In addition, each child’s weight and height were measured using standardized procedures and calibrated equipment, in accordance with international anthropometric protocols. All interviews and measurements were conducted at the Child Care and Development Center in a private setting to ensure confidentiality. Data collection was performed by trained and standardized personnel who were blinded to the study hypothesis. The field coordinator reviewed all data forms daily to verify completeness and internal consistency before digital entry into the study database.

### 2.3. Child Neurodevelopment

The neurodevelopmental assessment was conducted using the screening version of the Battelle Developmental Inventory (Battelle ID) and was administered by previously trained nursing students. Before data collection, all assessors completed a training program and a pilot assessment phase to ensure consistency and minimize inter-rater variability during administration.

This instrument evaluates fundamental developmental skills in children between the ages of birth and 8 years. Administering the test takes between 10 and 30 min. The test consists of 96 items grouped into five main areas: (1) Personal/social, (2) Adaptive, (3) Motor (gross and fine), (4) Communication (expressive and receptive), and (5) Cognitive. The assessment was conducted through a structured exam using an application booklet and supporting teaching materials. The children’s scores on the different items were then assessed. An informative interview was also conducted with their teachers based on the same application booklet. The instrument assesses whether the children are 1, 1.5, and 2 standard deviations below the mean. If the value is observed 1.5 standard deviations below the mean, it is considered sufficiently low to allow us to affirm that the child has a deficit. For the analysis in this study, standard deviations were used to construct the dependent variable; two categories were considered: 0 = no lag (1 standard deviation below the mean) and 1 = with lag (1.5 or 2 standard deviations below the mean). This categorization was applied to each neurodevelopmental area evaluated by the Battelle ID.

### 2.4. Overweight or Obesity

Weight and height measurements were performed by trained personnel using international techniques with previously calibrated instruments [21]. Weight (kg) and height (cm) were measured with participants standing, arms at their sides, without shoes and with as little clothing as possible. Based on these measurements, age- and sex-adjusted z-scores for body mass index (BMI) were calculated for the total sample. They were classified according to the World Health Organization as normal weight (BMI z score ≤ 1) and overweight or obese (BMI z score > 1), a category encompassing children at risk of overweight, those who are overweight, and those with obesity, to cover the entire spectrum of elevated adiposity) [22]. All measurements were performed in duplicate.

### 2.5. Statistical Analysis

BMI, neurodevelopmental scores, and general population characteristics were described as medians and interquartile ranges for continuous variables, and as absolute and relative frequencies for categorical variables. Before bivariate analysis, the distribution of continuous variables was assessed for normality using the Shapiro–Wilk test. Comparisons between groups were performed using the Mann–Whitney U test for continuous variables and Pearson’s χ^2^ test for categorical variables.

Multivariate analyses were conducted using independent logistic regression models for each neurodevelopmental domain. Potential confounders were identified based on prior literature and by applying the change-in-estimate criterion: variables whose exclusion from a saturated model resulted in a >10% change in the exposure effect estimate were retained [23]. The final models included the child’s sex, gestational age at delivery, birth weight, mother’s age, educational level, employment status, marital status, and number of children.

To evaluate the potential modifying effect of the child’s sex and maternal age on the association between each neurodevelopmental domain and BMI, multiplicative interaction terms (BMI × child’s sex; BMI × maternal age) were incorporated into the adjusted models [24]. Model specification, goodness of fit, and multicollinearity were examined across all models using standard diagnostic tests, including the variance inflation factor (VIF).

Statistical significance for both bivariate and multivariate analyses was defined as a two-tailed *p*-value ≤ 0.05. All analyses were conducted using Stata version 15.1 (StataCorp, College Station, TX, USA).

## 3. Results

The general characteristics of the study population are presented in Table 1. The median age of children was 59 months, the birth weight of girls was significantly lower than that of boys (3250 vs. 3120; *p* = 0.03), and 19.8% of the children participating were overweight or obese. Regarding maternal characteristics, approximately 50% of the children’s mothers were over 30 years old and had completed high school or less; we observed higher levels of education among mothers of girls compared to those of boys; more than 70% of the mothers reported having a partner, and more than 80% were employed.

**Figure 1:**
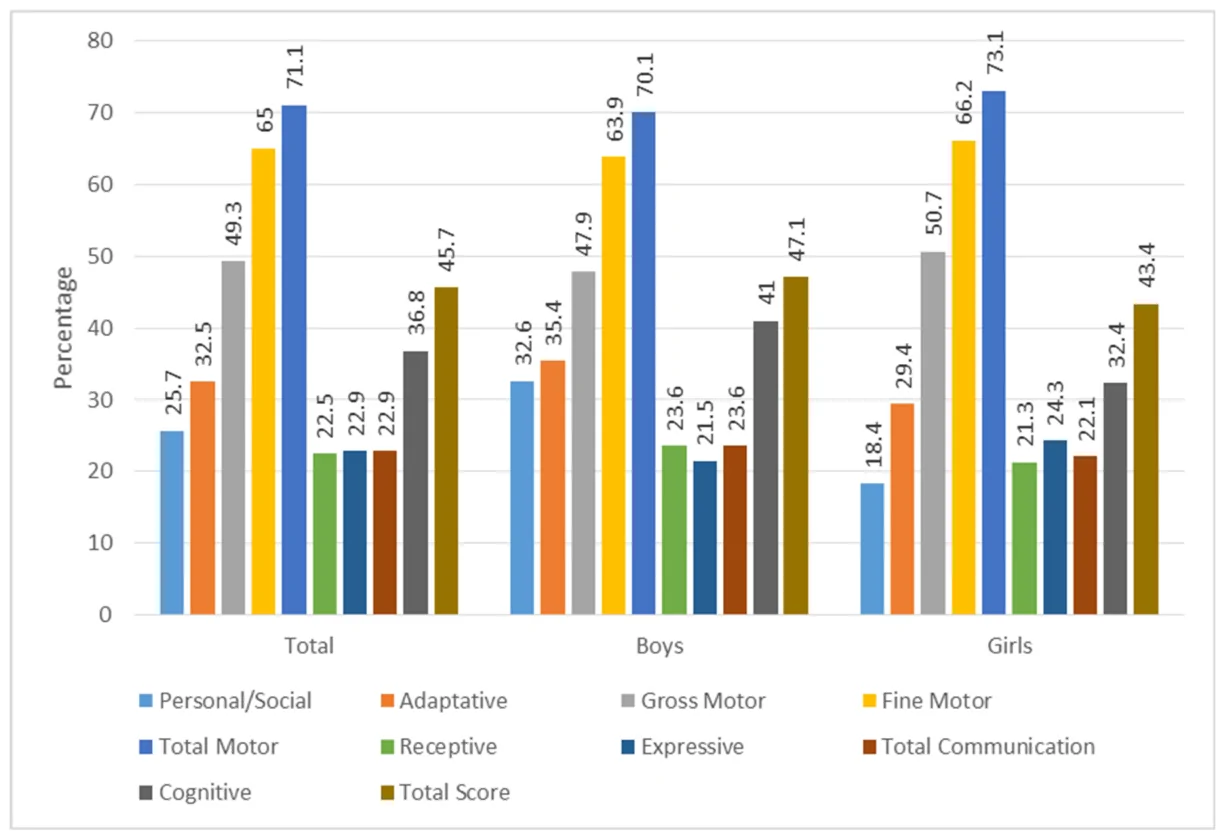
Percentage of children with neurodevelopmental delay.

In both boys and girls, more than 20% of children presented delays in each of the neurodevelopmental areas assessed (Figure 1). Regarding associations, we observed that those children who were overweight or obese were more likely to present neurodevelopmental delays in the Personal/Social Area [OR = 2.36 (95% CI 1.04–5.31)], and no significant associations were found in the other areas of neurodevelopment (Table 2).

We found significant interactions between overweight or obesity and the mother’s age (Adaptive Area *p* = 0.02) and overweight or obesity and the child’s sex (Personal/Social Area *p* = 0.001 and Communication Area *p* = 0.009); when evaluating the association in the models stratified by these variables, we observed a risk association in children of mothers under 30 years of age in the Adaptive Area [OR = 4.85 (95% CI 1.22–19.2)]; on the other hand, in the group of males with overweight or obesity, a greater odds of presenting delay in the Personal/Social and Communication Areas was observed [OR = 11.12 (95% CI 3.04–40.6) and OR = 5.88 (95% CI 1.63–21.2)] (Table 3).

## 4. Discussion

Our results suggest an association between overweight or obesity and delayed child neurodevelopment, specifically in the Personal/Social domain, which partially supports our initial hypothesis. Furthermore, significant interactions were identified between nutritional status and specific characteristics, such as the child’s sex and maternal age.

It was observed that children who were overweight or obese, as well as those whose mothers were 30 years old or younger at the time of the study, were more likely to experience delays in specific areas.

In our study, 19.8% of the children were overweight or obese, a proportion notably higher than the 7.7% reported nationally by the National Health and Nutrition Survey 2020–2023 (ENSANUT 2020–2023) [25]. This finding is alarming given the health problems associated with this condition. When interpreting this comparison, methodological differences must be taken into account: ENSANUT assessed BMI using a strict cutoff of +2 standard deviations, whereas our study evaluated BMI-for-age parameters—including children at risk of overweight (>+1 SD)—to detect early anthropometric vulnerability.

Furthermore, more than 20% of children presented delays in some neurodevelopmental areas, a result similar to that reported by ENSANUT 2021-2023, which found that 80.6% of children had adequate early childhood development (ECD) [26]. The Early Childhood Development Index (ECDI) assesses domains such as gross and fine motor skills, language, and social–emotional abilities, among others, which reflect how children function in daily activities and the competencies they acquire over time [27]. These domains closely correspond to those evaluated in our study. Similarly, previous research in Latin American populations has reported developmental delay prevalence ranging from 13.5% to 29.8% [7,28,29], which are comparable to the proportions observed in our sample.

When we evaluated the association between overweight or obesity and the different domains of neurodevelopment, we observed that children with excess weight were more likely to present delays in the Personal/Social domain. These findings are consistent with those reported in previous studies [30]. Nutritional status plays a key role in an individual’s social development [31]. Several studies conducted among school-aged children and adolescents have shown that obesity is associated with low self-esteem and poorer relationships with peers [32]; additionally, reports of discrimination by classmates toward children with obesity in school environments suggest that such experiences may negatively affect the development of social skills [32]. Similarly, another study found that children with normal body weight exhibited more positive social interactions than those with obesity [33].

With respect to the other domains assessed by the Battelle Developmental Inventory (Battelle ID), we did not find statistically significant associations with overweight or obesity; however, in nearly all domains, the direction of the associations was consistent with previous reports [34].

Regarding the motor domain, previous studies have suggested that excess body weight may restrict movement and thereby impair motor development [34,35,36]. For example, an analysis of 4- to 6-year-old children revealed that those with overweight or obesity showed fewer gross motor skills, such as jumping, compared to their normal-weight peers [35]. This association may be explained by a direct relationship between excess weight and impaired musculoskeletal function in affected children [37]. Consistent with our findings, the authors did not observe significant associations with fine motor skills [35]. This may be because fine motor skills develop gradually throughout childhood, and delays in this domain may become evident later as these skills become more complex [35].

Several authors have reported an inverse relationship between body mass index (BMI) and cognitive development in children, with higher BMI being associated with poor executive function performance [34,38]. Similarly, findings from a systematic review support an association between obesity among children and adolescents and deficits in executive function [38]. Although the underlying mechanisms remain unclear, it has been proposed that insulin and leptin (hormones involved in appetite and satiety regulation) also play a key role in brain plasticity, which could explain the observed associations [39,40].

Furthermore, our results suggest that overweight or obese children whose mothers were 30 years of age or younger were more likely to exhibit delays in adaptive development. Although this interaction has been scarcely explored, previous studies indicate that children’s adaptive development is strongly shaped by the environments in which they are raised and by the parenting strategies employed by their mothers [40]. These strategies, along with dietary habits that predispose to obesity, may vary by maternal age and level of parenting experience [40,41].

Likewise, it has been suggested that postponing motherhood allows women to attain higher levels of education, develop professional careers, and acquire life experiences that help create safe, healthy, and stimulating environments for their children [42]. Adaptive development encompasses the set of skills and abilities that enable a child to function independently in daily life. During childhood, acquiring these skills is essential, as it fosters autonomy in tasks such as eating, dressing, personal responsibility, and hygiene [43]. Therefore, the mother’s role is fundamental in promoting this aspect of development.

However, we found a significant interaction between the presence of overweight or obesity and sex in relation to delays in the Personal/Social and Language domains. Specifically, the results suggest that boys with obesity are more likely to exhibit delays in these areas.

These results are consistent with previous evidence showing that neurodevelopmental delays occur predominantly among boys [8,44]. Likewise, other studies have reported that obese male participants tend to be particularly affected in the social and language domains [30,45]. The mechanisms underlying this higher susceptibility remain unclear; therefore, further research is needed to elucidate the biological and environmental pathways that may explain this sex-specific association.

### 4.1. Strengths

To date, few studies have explored the association between excess-related nutritional problems and childhood neurodevelopment, making this study an essential contribution to understanding this relationship. We used multivariable models adjusted for potential confounders, thereby reducing the risk of residual confounding. Furthermore, all anthropometric and neurodevelopmental measurements were performed by trained personnel following standardized protocols and using calibrated equipment, which helps ensure measurement reproducibility and data reliability, while also minimizing the likelihood of measurement bias or classification error.

### 4.2. Limitations

This study has some limitations that should be considered when interpreting the results. First, because of the cross-sectional design, it is not possible to establish causal relationships among the variables analyzed. Second, given that participants were recruited from a single educational institution, the generalizability of our findings to other geographic or socioeconomic contexts may be limited. However, our results provide valuable evidence regarding the association between these variables; therefore, multicenter studies are recommended to allow for the generalizability of these findings. Third, it was not possible to include information on dietary habits, physical activity, sleep patterns, and screen time in the analysis; therefore, residual confounding in the observed associations cannot be ruled out.

Additionally, given that the sample size (n = 276) was calculated to detect effects of moderate magnitude (OR = 2.0), the study may have lacked sufficient statistical power to identify smaller-magnitude associations in the other neurodevelopmental domains. Consequently, the possibility of a Type II error regarding these specific results cannot be ruled out, highlighting the need for future research with larger samples.

Furthermore, the stratified analyses yielded relatively wide confidence intervals, likely due to the small sample size in specific strata. Consequently, these findings should be interpreted with caution, and future studies with larger strata are recommended to confirm these potential modifying effects.

## 5. Conclusions

Our results suggest that nutritional disorders, such as overweight and obesity, are associated with lower neurodevelopmental performance during early childhood, specifically in the personal–social domain. Furthermore, factors such as the child’s sex and maternal age may modify this association. While nutritional issues represent a major global public health challenge due to their link to chronic diseases, our findings indicate that their influence may also extend to specific dimensions of child development, particularly the acquisition of personal and social skills. These results highlight that childhood overweight or obesity may be significant not only for the development of physical comorbidities but also for essential aspects of human capital formation.

## Figures and Tables

**Table 1:** General characteristics of the study population.

Characteristics	Total (n = 280)	Boys (n = 144)	Girls (n = 136)	*p*-Value *
***Maternal***				
***Age***				
*≤30 years (%)*	41.5	42.3	40.7	
*>30 years (%)*	58.5	57.6	59.3	0.76
**Education (%)**				
High school or less	49.3	58.4	39.9	**0.006**
Bachelor’s degree	43.8	35.6	52.4	
Postgraduate	6.9	6.0	7.7	
**Marital status (%)**				
Without partner	28.7	30.4	26.9	0.48
With partner	71.3	69.6	73.1	
**Employment status (%)**				
No	16.6	19.6	13.4	0.14
Yes	83.4	80.4	86.6	
***Children***				
**Type of delivery (%)**				
Vaginal	46.4	41.4	52	0.10
Cesarean	53.6	58.6	48	
**Place of delivery (%)**				
Private hospital	32.4	34.6	30	0.40
Public hospital	67.6	65.4	70	
**Birth weight (g)**	3200 (595)	3250 (550)	3120 (600)	0.03 **
**Gestational age at birth (weeks)**	39 (2)	39 (2)	39 (1)	0.42 **
**Child’s age (months)**	59 (15)	58 (15)	59 (15)	0.55 **
**Nutritional status**				
**BMI (%)**				
Normal weight	80.2	81.3	79	0.62
Overweight or obesity	19.8	18.7	21	

Values for continuous variables are medians (interquartile range) and for categorical variables, percentages. * *p*-value from Pearson’s χ^2^ test. ** *p*-value from Mann–Whitney U test. Statistical significance: *p* ≤ 0.05.

**Table 2:** Odds ratios of the association between neurodevelopmental areas and the presence of overweight or obesity.

Neurodevelopmental Area	OR	95% CI	*p*-Value
**Personal/Social Area**			
Overweight or Obesity	2.36	1.04–5.31	**0.03**
**Adaptive Area**			
Overweight or Obesity	1.33	0.61–2.89	0.47
**Motor Area**			
Overweight or Obesity	1.05	0.47–2.33	0.89
**Fine Motor**			
Overweight or Obesity	0.62	0.29–1.32	0.21
**Gross Motor**			
Overweight or Obesity	1.39	0.66–2.92	0.37
**Communication Area**			
Overweight or Obesity	1.88	0.76–4.63	0.16
**Receptive**			
Overweight or Obesity	1.00	0.39–2.57	0.99
**Expressive**			
Overweight or Obesity	1.23	0.51–2.98	0.64
**Cognitive Area**			
Overweight or Obesity	1.08	0.51–2.29	0.83
**Total score**			
Overweight or Obesity	1.10	0.53–2.29	0.79

Adjusted for child’s age, child’s sex, weeks of gestation, mother’s age, mother’s education, mother’s marital status, mother’s employment status, number of children, and birth weight. The reference category was Normal Weight.

**Table 3:** Odds ratios of the association between neurodevelopmental areas and the presence of overweight or obesity stratified by mother’s age or child’s sex.

Neurodevelopmental Area	OR	95% CI	*p*-Value	Interaction *p*-Value
**Adaptive Area** **^a^**				
Overweight or obesity × Maternal age ≤ 30 years	4.85	1.22–19.2	**0.02**	**0.02**
Overweight or obesity × Maternal age > 30 years	0.57	0.18–1.80	0.34	
**Personal/Social Area** **^b^**				
Overweight or obesity × Boys	11.12	3.04–40.6	**<0.0001**	**0.001**
Overweight or obesity × Girls	0.32	0.05–1.75	0.19	
**Communication Area** **^b^**				
Overweight or obesity × Boys	5.88	1.63–21.23	**0.007**	**0.009**
Overweight or obesity × Girls	0.42	0.08–2.18	0.30	

^a^ Adjusted for child’s age, child’s sex, weeks of gestation, mother’s education, mother’s marital status, mother’s employment status, number of children, and birth weight. ^b^ Adjusted for child’s age, mother’s age, weeks of gestation, mother’s education, mother’s marital status, mother’s employment status, number of children, and birth weight. The reference category was Normal Weight.

## Data Availability

The original data presented in the study are openly available in Mendeley Data at https://doi.org/10.17632/zhtbkx6wdj.1 (accessed on 6 June 2026).

## Abbreviations

The following abbreviations are used in this manuscript:

Batelle ID	Battelle Developmental Inventory
BMI	Body Mass Index
CI	Confidence interval
ECD	Early Childhood Development
ECDI	Early Childhood Development Index
ENSANUT	National Health and Nutrition Survey
OR	Odds Ratio
VIF	Variance Inflation Factor
WHO	World Health Organization
